# Negative Stress Beliefs Predict Somatic Symptoms in Students Under Academic Stress

**DOI:** 10.1007/s12529-016-9562-y

**Published:** 2016-04-18

**Authors:** Susanne Fischer, Urs M. Nater, Johannes A. C. Laferton

**Affiliations:** 1 Department of Psychology, Clinical Biopsychology, University of Marburg, Gutenbergstr. 18, 35032 Marburg, Germany; 2 Psychological Medicine, King’s College London, Institute of Psychiatry, Psychology & Neuroscience, 103 Denmark Hill, PO74, London, SE5 8AF UK; 3 Department of Psychology, Clinical Psychology & Psychotherapy, University of Marburg, Gutenbergstr. 18, 35032 Marburg, Germany

**Keywords:** Beliefs about stress, Medically unexplained symptoms, Negative expectations, Somatic symptoms, Stress

## Abstract

**Purpose:**

Medically unexplained symptoms are abundantly present in the general population. Stress may lead to increased symptom reporting because of widespread beliefs that it is dangerous for one’s health. This study aimed at clarifying the role of stress beliefs in somatic symptom reporting using a quasi-experimental study design.

**Methods:**

Two hundred sixteen German university students (60 % of an initial sample of 363) were examined at the beginning of the term (less stressful period) and at the end of the term (stressful period due to exams). Negative beliefs about stress at baseline were expected to predict somatic symptoms at follow-up.

**Results:**

Negative beliefs about stress at baseline significantly predicted somatic symptoms at follow-up (*β* = 0.16, *p* = .012), even when controlling for general strain, physical and mental health status, neuroticism, optimism, and somatosensory amplification.

**Conclusions:**

Being convinced that “stress is bad for you” was prospectively associated with somatic symptoms during a stressful period. Further research in patients with medically unexplained conditions is warranted to corroborate these findings.

## Introduction

Somatic symptoms are abundantly reported in the general population and at different levels of health care systems [e.g., 1]. While medical diseases account for many somatic symptoms, there is a surprisingly large proportion of patients [over 35 %; 2, 3] whose complaints remain “medically unexplained.” The last decades have witnessed a shift towards a multi-factorial explanation of medically unexplained symptoms. One of the most frequently discussed pathogenic factors is stress, as it has been found to be involved in the predisposition, precipitation, and perpetuation of numerous medically unexplained conditions, such as chronic fatigue syndrome or fibromyalgia syndrome [4].

Stress is a ubiquitous and frequently discussed phenomenon in modern societies. From a scientific point of view, stress occurs whenever a situation is perceived as threatening and one’s means of dealing with it are deemed insufficient [5]. This kind of appraisal results in an emotional, behavioral and biological “stress response.” The situations that lead to such a response are labelled “stressors.” Importantly, *acute* stress is considered adaptive, whereas it is well-known that *chronic* stress may involve a wear and tear of stress-responsive bodily systems, such as the hypothalamic-pituitary-adrenal axis or the autonomic nervous system [6]. As such, chronic stress has been associated with numerous medical diseases and mental disorders [7], including medically unexplained conditions [8].

However, lay persons may have a different understanding of stress and how it may affect health. For instance, in a qualitative study on workplace stress by Kinman and Jones [9], more than half of the sample did not distinguish between stressors and stress responses. In addition, although the overall sample held rather nuanced views about stress, 16 % viewed occupational stress as wholly detrimental. It is therefore conceivable that stress may also lead to medically unexplained symptoms because of beliefs that stress is dangerous for one’s health [10]. According to this notion, medically unexplained symptoms would develop when a significant amount of attention is being paid to transient bodily sensations, when these are interpreted as signs of a pathogenic process, and subsequently labelled as “symptoms.” However, this hypothesis has so far never been tested.

In behavioral medicine, beliefs about stress have only recently captured researchers’ attention. Keller et al. [11] were the first to show that negative stress beliefs were common (about one third of their community sample held such beliefs) and that they were associated with morbidity and mortality. Similarly, another study showed that negative stress beliefs predicted myocardial infarction and coronary death in the Whitehall II cohort [12]. However, in both of these studies, participants were asked whether they believed that stress had affected their health in the past. The measures thus exclusively referred to past experience and did not ask about participants’ current beliefs. Nothing is known about whether viewing stress as something negative is prospectively associated with ill health. It equally remains unclear whether positive beliefs about stress and the extent of which an individual feels in control of his stress levels are linked with good health.

The primary aim of this study was to investigate for the first time whether negative stress beliefs would predict somatic symptom reporting. We chose to measure beliefs about stress during a less stressful period (*T*_0_), with a follow-up assessment of somatic symptoms during a stressful period (*T*_1_). The approach of this study was thus to scrutinize the stress belief-ill health association in a quasi-experimental fashion. A second goal was to explore whether positive beliefs about stress and perceived control over stress would have a protective effect, that is, whether scoring high on these two variables would predict stability or even a decrease in the intensity of somatic symptoms. Finally, we aimed at exploring potentially mediating factors in the presumed relationship between beliefs about stress and somatic symptoms, such as additionally increased/decreased stress levels during academic exams. We decided to recruit a student sample for the present study, which was expected to mainly consist of young, healthy individuals with a high socioeconomic status. An advantage of this approach is that it enables the exploration of stress beliefs and somatic symptoms in a homogenous sample that is free of major confounding influences. For instance, it is conceivable that past experiences with severe illnesses or the presence of a chronic illness may affect both stress beliefs and the general level of somatic symptoms. Another relevant advantage for this study was the fact that students have common stress experiences, for example when they undergo academic exams.

## Methods

### Recruitment

All students of the University of Marburg (Germany) were invited to take part in the study via e-mail. The only eligibility criteria were being a student and having academic examinations at the end of the summer term (stressful condition). *N* = 363 provided baseline data, and *N* = 216 provided both baseline and follow-up data (60 % retention rate). The final sample size for statistical analysis was therefore *N* = 216. Participation was voluntary and informed consent was obtained from all individual participants included in the study. All procedures were in accordance with the ethical standards laid down in the Declaration of Helsinki, and the study was approved by the local ethics committee.

### Study Design

The participants took part in two consecutive surveys: one close to the beginning of the summer term (less stressful period, *T*_0_) and one around the end of the summer term, while undergoing academic examinations (stressful period, *T*_1_). Beliefs about stress, stress levels, baseline somatic symptom intensity, health status, and a number of potentially confounding traits (see below) were assessed at baseline. The follow-up survey was shorter and as such merely focused on stress levels and somatic symptom intensity. Importantly, the first assessment period started 4 weeks into the summer term, which is when we assumed students would have fully transitioned back into their academic routines and stress levels would therefore be lowest. The exact dates of sending out the invitations for the second survey were tailored to individual examinations days, but usually took place 6 to 8 weeks after the initial assessment (i.e., in the final weeks of the term, which lasts around 12 weeks at the University of Marburg, or shortly after it had ended).

### Measures

*Stress* levels were measured twice to check whether the presumed “stressful period” (*T*_1_, when students had their examinations) was in fact more stressful than the baseline period (*T*_0_, when the term had just started) and to test whether increased/decreased stress levels were a mediating factor in the presumed beliefs-somatic symptoms relationship. The twelve item Screening Scale for the Assessment of Chronic Stress [SSCS; 13] was used for this purpose. Importantly, the scale only referred to the past 2 weeks. It covers different aspects of stress, such as worrying, work and social overload, excessive demands at work, and lack of social recognition. The answering scale uses five options ranging from “does not apply at all” (0) to “strongly applies” (4).

*Somatic symptoms* were measured twice by means of a combination of two instruments: the Generic Assessment of Side Effects [GASE; 14] and a German translation of the Subjective Health Complaints Inventory [SHC; 15]. A total of 26 somatic symptoms were included into the analysis: having a cold, muscular tension, back pain, headache, migraine, chest pain, breathing problems, sleep problems, fatigue, racing heart, dizziness, circulation problems, tinnitus, abdominal pain, gas discomfort, diarrhea, constipation, difficulty urinating, heartburn, nausea, allergies, eczema, hair loss, loss of appetite, increased appetite, and painful or irregular menstruation (women only). Participants rated the intensity of each complaint on a four point Likert scale, from “not present” (0) to “mild” (1), “moderate” (2), and “severe” (3), again with regards to the past 2 weeks.

*Beliefs about stress* were measured via the newly developed and recently validated Beliefs about Stress Scale (BASS, Laferton & Fischer, under revision). The questionnaire measures negative stress beliefs (eight items) and positive stress beliefs (four items) as well as perceived control (three items). Items were generated based on the stress beliefs literature, interviews with lay persons, and already existing questionnaires measuring related constructs [16, 17]. Example items are as follows: “Being stressed affects my health in the short-term” (negative belief), “Being stressed enables me to work in a more focused manner” (positive belief), or “Being stressed is something I am able to influence through my actions” (perceived control). The answering scale uses four options ranging from “completely disagree” (1) to “definitely agree” (4). In the above mentioned validation study, internal consistency was *α* = .80 for negative stress beliefs, *α* = .87 for positive stress beliefs, and *α* = .73 for perceived control.

*Covariates* were measured using a variety of well-established questionnaires. The presence of general (including non-academic) strain was assessed via a dichotomous item of the Patient Health Questionnaire [18]. Physical and mental health was assessed via a single dichotomous item (“Have you been diagnosed with a medical disease or mental disorder?”; no equaled 0, yes equaled 1). Neuroticism was measured by means of the ten item Big Five Inventory [19]. Optimism was assessed via the ten item Life Orientation Test [20]. Somatosensory amplification, that is, a cognitive style which is characterized by experiencing somatic sensations as more intense and evaluating them as more negative, was measured by the ten item Somatosensory Amplification Scale [21].

### Statistical Analysis

A *t* test for paired samples was performed to check whether students were in fact more stressed and had more severe somatic symptoms during the presumed “stressful period” (*T*_1_), when compared to the baseline period (*T*_0_). Next, a hierarchical regression was calculated to evaluate whether beliefs about stress as measured at baseline (second block) predicted somatic symptoms at *T*_1_ above and beyond baseline somatic symptoms, general strain, physical and mental health problems, and neuroticism, optimism (reversed scoring), and somatosensory amplification scores (first block). In addition, it was tested whether a potential relationship between negative beliefs about stress and somatic symptoms at *T*_0_ would predict somatic symptoms at *T*_1_ (interaction term, third block). Finally, provided the expected relationship between stress beliefs and somatic symptoms was confirmed, an explorative analysis was planned to test whether this was due to an additional increase in stress levels at *T*_1_. We used the SPSS 21 (Chicago, IL) and the PROCESS macro developed by Andrew F. Hayes (http://www.processmacro.org) to conduct mediation analyses. Only work-related SSCS items were included into mediation analysis, as we were specifically interested in testing whether the beliefs predicted an increase in academic rather than general stress levels.

## Results

### Sample Characteristics

Our sample consisted of 156 women (72.2 %) and 60 men (27.8 %), and mean age was 23.12 ± 2.83 (SD). Students had been enrolled for 5.24 ± 3.22 semesters on average and most frequently studied arts, followed by social and natural sciences. Their stress levels (SSCS) at baseline were slightly above population-based norm values, and there was a small but significant increase from *T*_0_ to *T*_1_ (22.76 ± 9.26 vs. 24.36 ± 9.15; *t*(215) = −3.29, *p* = .001). This means that the academic examinations did in fact act as a mild natural stressor in this study. The intensity of students’ baseline somatic symptom reporting (combined GASE and SHC) was at 16.6 ± 10.4 out of a maximum attainable score of 78, again with slightly increased levels at follow-up (17.4 ± 9.9; *t*(215) = −1.51, *p* = .134). Baseline mean values and standard deviations, or relative frequencies of the remaining variables can be found in Table 1.

**Table 1:** Descriptives of study variables (*N* = 216)

Variable (theoretical scale range)	M ± SD, relative frequency
Negative stress beliefs^a^ (8–32)	23.3 ± 4.5
Positive stress beliefs^a^ (4–16)	10.3 ± 3.0
Perceived control^a^ (3–12)	8.4 ± 1.8
Presence of general strain^b^ (0 vs. 1)	46.3 %
Presence of physical/mental illness^c^ (0 vs. 1)	24.5 %
Neuroticism^d^ (2–10)	6.7 ± 2.0
Optimism^e^ (0–24)	14.7 ± 4.5
Somatosensory amplification^f^ (10–50)	27.3 ± 6.0

^a^Beliefs about Stress Scale

^b^Patient Health Questionnaire (single dichotomous item)

^c^Single dichotomous item

^d^Big Five Inventory (10 item version)

^e^Life Orientation Test (10 item version)

^f^Somatosensory Amplification Scale (10 item version)

### Beliefs About Stress as Predictor of Somatic Symptoms

All assumptions for conducting a hierarchical regression were met. Among our covariates (first block), the number of baseline somatic symptoms was the only significant predictor of follow-up symptoms (*β* = 0.71, *p* < .001). Together with general strain, physical and mental health status, neuroticism, optimism, and somatosensory amplification, it explained 54 % of total variance (*p* < .001). Furthermore, in line with our expectations, negative beliefs about stress at baseline (second block) significantly predicted more intense somatic symptoms during the stressful period (*β* = 0.16, *p* = .012; see also Table 2). No such associations emerged regarding baseline positive beliefs (*β* = −0.00, *p* = .967) and perceived control (*β* = 0.01, *p* = .847). Beliefs about stress explained 2 % of additional variance in somatic symptoms at *T*_1_ (*p* = .039). Including the relationship between negative stress beliefs and somatic symptoms at *T*_0_ into the model (interaction term, third block) did not lead to a significant increase in explained variance, and the term was not found to be a significant predictor of somatic symptoms at *T*_1_ (*β* = 0.13, *p* = .631).

**Table 2:** Hierarchical regression predicting the intensity of somatic symptoms at follow-up (*T*_1_; *N* = 216); covariates were entered at step 1, beliefs about stress at step 2, and an interaction term including negative beliefs about stress and baseline somatic symptoms at *T*_0_ was entered at step 3

Model	Predictor	*β*	R^2^	R^2^ adj.	ΔR^2^
1			0.54	0.53	***0.54
	Somatic symptoms at *T*_0_^a^	***0.71			
	General strain^b^	0.02			
	Physical/mental illness^c^	−0.00			
	Neuroticism^d^	0.02			
	Optimism^e^	−0.02			
	Somatosensory amplification^f^	0.02			
2			0.56	0.54	*0.02
	Somatic symptoms at *T*_0_^a^	***0.70			
	General strain^b^	0.03			
	Physical/mental illness^c^	0.01			
	Neuroticism^d^	−0.02			
	Optimism^e^	0.01			
	Somatosensory amplification^f^	−0.01			
	Negative stress beliefs^g^	*0.16			
	Positive stress beliefs^g^	−0.00			
	Perceived control^g^	0.01			
3			0.56	0.54	0.00
	Somatic symptoms at *T*_0_^a^	*0.58			
	General strain^b^	0.04			
	Physical/mental illness^c^	0.01			
	Neuroticism^d^	−0.01			
	Optimism^e^	0.01			
	Somatosensory amplification^f^	−0.01			
	Negative stress beliefs^g^	0.12			
	Positive stress beliefs^g^	−0.00			
	Perceived control^g^	0.01			
	Negative beliefs*symptoms at *T*_0_	0.13			

*p < .05, **p < .01, ***p < .001

^a^Generic Assessment of Side Effects and Subjective Health Complaints Inventory

^b^Single dichotomous item of the Patient Health Questionnaire

^c^Single dichotomous item

^d^Big Five Inventory

^e^Life Orientation Test

^f^Somatosensory Amplification Scale

^g^Beliefs about Stress Scale

We then explored whether negative stress beliefs predicted the number of somatic symptoms via an additional increase in stress during the examination period. Indeed, the indirect effect proved significant (*β* = 0.15, *p* < .001) and corresponded to a medium effect size [22].

## Discussion

In this study, having strong negative stress beliefs at baseline predicted somatic symptom reporting during a stressful period (academic examinations). Importantly, this was true even when accounting for the general amount of somatic symptoms students experienced, which is one of the strongest predictors of incident medically unexplained conditions [23].

Being convinced that “stress is bad for you” may therefore lead to a higher somatic symptom load when stress levels increase. As pointed out by Keller et al. [11], this process resembles a self-fulfilling prophecy. Potentially underlying mechanisms are beginning to be unraveled by recent advances in nocebo research, which posits that symptoms worsen as a consequence of negative expectations [24]. The brain reward circuitry as well as anxiety and subsequent activation of both the cholecystokinin (CCK) and hypothalamic-pituitary-adrenal (HPA) systems seem crucial facilitators of these effects. This is interesting in light of the fact that in the current study, increased stress levels (which are associated with HPA axis activation) partially mediated the stress beliefs-somatic symptoms relationship.

One limitation of the present study is the use of a student sample, which mainly consisted of young, healthy women with a high socioeconomic status. This limits the generalizability of the findings to the general population, although the female preponderance seems less problematic seeing that women are more frequently affected by medically unexplained conditions [23]. As a next step, it would be interesting to study the frequency of negative and positive stress beliefs among people with various educational backgrounds. Another limitation concerns the fact that the natural stressor (academic exams) only led to a mild increase in stress levels and somatic symptoms in the current sample. Future research may therefore consider using experimental designs that may allow for a more effective manipulation of stress levels. This may also lead to a stronger effect of negative stress beliefs on somatic symptoms, compared to the small effect observed in this study.

According to Brown [25], negative expectations may be relevant to all kinds of medically unexplained conditions. As direct verbal suggestion is likely to be the major cause of negative stress beliefs, clinicians need to be aware of how they inform patients about the role of stress in health and disease. A recent experimental study showed that learning to appraise stress-induced bodily sensations as positive “arousal cues” (instead of disease warning signs) led to more beneficial acute cardiovascular stress responses [26]. Further research is warranted to corroborate the findings of the present study in patient samples and to pin down the mechanisms translating stress beliefs into medically unexplained conditions. To this end, experimental studies measuring the psychobiological response to a combination of negative verbal suggestion and stress may prove informative.
